# Facile Fabrication of an Ammonia-Gas Sensor Using Electrochemically Synthesised Polyaniline on Commercial Screen-Printed Three-Electrode Systems

**DOI:** 10.3390/s21010169

**Published:** 2020-12-29

**Authors:** Anja Korent, Kristina Žagar Soderžnik, Sašo Šturm, Kristina Žužek Rožman, Nathalie Redon, Jean-Luc Wojkiewicz, Caroline Duc

**Affiliations:** 1 Department for Nanostructured Materials, Jožef Stefan Institute, Jamova cesta 39, SI-1000 Ljubljana, Slovenia; kristina.zagar@ijs.si (K.Ž.S.); saso.sturm@ijs.si (S.Š.); tina.zuzek@ijs.si (K.Ž.R.); 2 Jožef Stefan International Postgraduate School, Jožef Stefan Institute, Jamova cesta 39, SI-1000 Ljubljana, Slovenia; 3 IMT Lille Douai, Institut Mines-Télécom, University of Lille, Centre for Environment and Energy, F-59000 Lille, France; nathalie.redon@imt-lille-douai.fr (N.R.); jean-luc.wojkiewicz@imt-lille-douai.fr (J.-L.W.); caroline.duc@imt-lille-douai.fr (C.D.)

**Keywords:** cyclic voltammetry (CV), electropolymerisation, NH_3_ detection, polyaniline (PANI), screen-printed electrode (SPE)

## Abstract

Polyaniline (PANI) is a conducting polymer, widely used in gas-sensing applications. Due to its classification as a semiconductor, PANI is also used to detect reducing ammonia gas (NH_3_), which is a well-known and studied topic. However, easier, cheaper and more straightforward procedures for sensor fabrication are still the subject of much research. In the presented work, we describe a novel, more controllable, synthesis approach to creating NH_3_ PANI-based receptor elements. The PANI was electrochemically deposited via cyclic voltammetry (CV) on screen-printed electrodes (SPEs). The morphology, composition and surface of the deposited PANI layer on the Au electrode were characterised with electron microscopy, Fourier-transform infrared spectroscopy and profilometry. Prior to the gas-chamber measurement, the SPE was suitably modified by Au sputtering the individual connections between the three-electrode system, thus showing a feasible way of converting a conventional three-electrode electrochemical SPE system into a two-electrode NH_3_-gas detecting system. The feasibility of the gas measurements’ characterisation was improved using the gas analyser. The gas-sensing ability of the PANI-Au-SPE was studied in the range 32–1100 ppb of NH_3_, and the sensor performed well in terms of repeatability, reproducibility and sensitivity.

## 1. Introduction

Conductive polymers have great potential as gas-sensing materials because of their light weight, low cost, and straightforward production, which are associated with the electronic, magnetic and optical properties of metals [1]. The π-conjugation along the chains of those materials allows the formation of a delocalised electron at the origin of the electrical conduction, which can be modulated by a redox reaction or protonation from a near-insulator to a metallic conductor [2,3]. Integrated into chemiresistive sensors for the detection of oxidising or reducing gases [4,5,6] (NH_3_ [6,7,8,9,10], hydrogen [11], carbon monoxide [10], nitrogen oxides (NO_x_) [7,12], and hydrogen sulphide [12]), they enable room-temperature (RT) measurements, i.e., much lower than the operating temperatures of metal oxides that are conventionally used in gas sensing and operate at temperatures ~300–400 °C [5]. Moreover, in comparison with metal oxides, they show good metrological characteristics, such as a high sensitivity and a short response time, combined with the ability to be easily tuned, both chemically and physically [13].

Detecting ammonia (NH_3_) at low concentrations is crucial in many environmental and biomedical situations. Firstly, NH_3_ is essential to various industries, e.g., chemical, medical, food, and automotive. Still, it can also be problematic concerning the environment and human health, i.e., it is highly reactive and can trigger skin, eye, and lung illnesses [6,14]. According to the Occupational Safety and Health Administration (OSHA) [15], the exposure limit for humans is 50 ppm for 8 h and 35 ppm for 15 min. Humans are incapable of sensing the presence of NH_3_ at concentrations below 5 ppm [16,17], and NH_3_ is highly reactive. It can form harmful, nanosized aerosols (particulate atmospheric matter such as ammonium nitrate and ammonium sulphate) [14]. Thus, it is essential to develop detection techniques that are quick to react, convenient and reliable for detecting NH_3_ in low concentrations. Secondly, NH_3_ has been reported to be a relevant marker for liver and kidney diseases [18]. In the case of these organs’ failure, the NH_3_ concentration from exhaled breath increases from a few hundred ppb to several ppm [19]. The detection of NH_3_ over such a broad concentration range in human breath shows promise as a non-invasive and low-cost technique for diagnosing and/or monitoring such diseases [20,21]. The ability to sense NH_3_ in a low-ppb range can be transferred to the indirect detection of other toxic gases, where NH_3_ is a by-product of a certain chemical reaction triggered by an applied organic compound or catalyst. For example, formaldehyde gas was detected by using polyaniline (PANI) as a sensing material for NH_3_ produced as a by-product of an organic reaction between formaldehyde and Fluoral-P [22,23].

Different working principles can be used to detect ammonia, such as electronics, electrochemistry, optical, surface acoustic wave and field-effect transistor. Optical methods such as tuneable-diode-laser adsorption spectroscopy have the highest sensitivity (150 ppt) and selectivity. However, their operating conditions do not match all the application requirements. For this reason, chemiresistive sensors are the most popular. Presenting sensitivities in the range 20 to 100 ppb, they have more cost-effective and simpler fabrication and integration [14,24].

Among the sensitive materials in chemiresistive sensors, PANI is one of the most studied conductive polymers for NH_3_ sensing [4]. It is based on the ability of the gas to change the resistance of the material via the well-known deprotonation/protonation mechanism [25,26]. It is, in addition, light weight, inexpensive, and environmentally stable with a straightforward polymerisation reaction that has high yields and reversible redox and pH-switching properties. Its conductivity can be adjusted by changing its oxidation and protonation state, which makes PANI unique in the family of conductive polymers. PANI can be found in various oxidative states, with three being the most known: the fully reduced state (leucoemeraldine), the half-oxidised state (emeraldine) and the fully oxidised state (pernigraniline). Emeraldine is the most conductive form of PANI [27]. Emeraldine has high stability at room temperature and is classified as a semiconductor, which makes it suitable for gas sensing [28]. However, like the other conductive polymers, PANI’s main limitations are a sensitivity to humidity and problems with long-term stability [14].

In order to enhance PANI;s conductivity, its switching abilities, and its morphological and structural properties that consequently influence the response to NH_3_ [5,6], many different formulations have been developed. They range from using pure PANI with simple dopants such as HClO_4_ [29] or polymers (polyurethane [30], poly(methyl methacrylate) [31]), in addition to more complex dopants (dodecylbenzenesulfonic acid (DBSA), camphorsulfonic acid (CSA)) [8] or nanomaterials such as carbon nanotubes [21,32,33] and metal particles [5,33] or metal oxides [9,34,35]).

**Figure 1:**
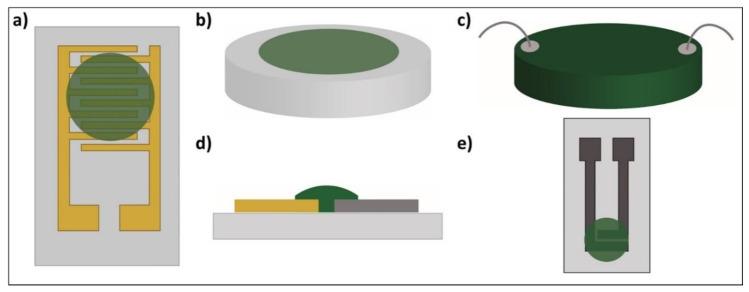
Polyaniline (PANI)-based ammonia/humidity gas sensors: (**a**) interdigitated electrodes [8,9,21,30,36]; (**b**) film [40]; (**c**) pellet [38]; (**d**) two different metal electrodes [39]; (**e**) screen-printed two-electrode system [37].

Moreover, various configurations have been used to monitor the changes in the PANI’s resistance [8,9,21,30,36,37,38] or impedance [39] in the presence of NH_3_ gas. They consist of (i) two-point resistivity measurements that are usually made using either PANI deposited on interdigitated electrodes with multiple “fingertips” [8,9,21,30,36] (Figure 1a), (ii) a PANI film on glass substrates [40] (Figure 1b), (iii) a PANI pellet connected by wires [38] (Figure 1c), (iv) a PANI film deposited on two electrodes made of different metals [39] (Figure 1d) or (v) PANI in a screen-printed two-electrode system [37] (Figure 1e). The deposition of PANI on such electrodes is usually completed with drop-casting [9,21,30,37,39], spin-coating [8,41] and layer-by-layer assembly [41]. In all these cases, PANI is synthesised via a chemical polymerisation method.

Knowing the impact of the PANI’s morphology and the PANI’s film thickness on gas-sensing performance [41,42], the processes of its synthesis and its deposition are thus crucial. In this respect, PANI’s electrochemical polymerisation allows easier control over the PANI’s film thickness and the morphology of the final product by controlling the parameters of the electrochemical deposition, such as deposition time, applied current or applied voltage. Indeed, the result of such a synthesis is a nanostructured polymer in conductive, doped form due to the polymerisation in an acid electrolyte [27,43]. Such a synthesised polymer is well integrated and compacted with the surface of the working electrode [44] that can be used for gas sensing.

As per the electrochemical synthesis principle, the required three-electrode system (the working, counter and reference electrodes) [27,43] allows the deposition of material only on the surface of the working electrode, which represents a significant drawback and challenge for resistance-based gas measurements. Paul et al. [45] electrodeposited PANI on an eight-electrode array with a 1.7 μm gap between the Au electrodes. Indeed, in the case of a sufficiently small gap (1–10 μm) and long polymerisation times, PANI can assemble over the gap [36,45,46,47], making it possible to construct two solid-state electrodes, in between which a conducting material is sandwiched. However, such an approach is difficult and expensive in terms of the demand of a particular small electrochemical cell, which needs to enable the contact of the electrolyte with a two-electrode system and a removable reference electrode. In the case of a three-electrode SPE, only a drop of electrolyte is needed, and when a conductive material is electrochemically deposited on the working electrode, for further functionalisation, only suitable contacts on the top of the deposited material still need to be made. These contacts must not be in contact with the working electrode, but must connect the deposited material with the secondary electrode [48]. The deposited material and the type of synthesis represent a bottleneck for these approaches.

By combining a knowledge of resistivity-based gas sensing and electrochemistry, we focused our work on developing a NH_3_-detection system based on electrochemically synthesised “pure” PANI without the use of any complex dopants [29,30,31] and nanomaterials [5,21,32,33,49,50]. PANI was chosen because of its superior sensing performance with regard to ammonia [6,51]. We report on a direct electrodeposition procedure for PANI on a commercial, screen-printed electrode (SPE) that results in the PANI′s excellent uniformity, and high reproducibility, with the potential for mass production and in-situ analyses [52]. Moreover, this procedure is suitable for automation processes, and has an increased reliability and repeatability. The proposed procedure enables the feasible production of low-cost sensor devices without the extensive use of chemicals/materials. A sensor prepared in this way was characterised in different humidity conditions and NH_3_ concentrations. In addition, by including a real-time analyser for NH_3_ gas, two different approaches for sensor-sensitivity characterisation were used. As a result, by using this inexpensive and practical fabrication procedure we were able to construct a robust, ppb-range NH_3_ sensor. Indeed, at room temperature and under 70% of humidity, the sensor in this study shows a limit of detection of 23 ppb and sensitivities of 12.30 and 4.27%.ppm^−1^ in the ranges 32–200 ppb and 200–1000 ppb, respectively.

## 2. Materials and Methods

### 2.1. Polyaniline Synthesis

PANI can be electrodeposited on a metal electrode using three different electrochemical techniques (potentiodynamic, potentiostatic, and galvanostatic). The potentiodynamic method (cyclic voltammetry, CV), exposes the growing polymer to additional processes, i.e., oxidation/reduction reactions, which adds another dimension to the electropolymer’s growth [27]. By changing the CV conditions (scan rate, number of cycles) the polymer’s properties can be tailored. By applying a slower scan rate (25, 50 mV/s) the peak potential of the first redox couple shifts towards lower potentials and the resulting polymer has a higher growth rate in comparison to the polymer grown with high scan rates (100 mV/s). It was shown that by using 50 mV/s the lowest average diameter of PANI nanofibers is achieved [53]. Controlling the charge consumed for electropolymerisation enables an estimation of the amount of deposited PANI. Only the first oxidation peak of the PANIs’ CV behaviour contains information of just the PANI reaction. In all the other peaks, pure bare-electrode material oxidation or reduction is included [53,54,55]. Therefore, by controlling the equality of the anodic current of the first oxidation, the reproducibility between polymerisations can be achieved. Based on this, the experimental conditions for the PANI’s polymerisation were chosen.

The PANI was electrodeposited on a commercial DropSens screen-printed electrode (SPE; MetrohmDropSens, ref. SPE-250AT) [56], with gold as the working electrode, platinum as the counter electrode and silver as the reference electrode. The electropolymerisation was carried out in an acidic medium (1 M HCl prepared from 37% HCl Sigma Aldrich, pH = 0.43) containing a 0.1 M aniline monomer (Sigma Aldrich). The electrochemical experiments were performed on a USB- and battery-powered potentiostat/galvanostat/EIS analyzer PalmSens4. CV, as the deposition method, was performed at a potential of −0.3 V to 1 V using a scan rate of 50 mV/s. The CV was stopped after completing the cycle in which the first oxidation peak reached an anodic current of 1.5 mA to achieve reproducibility for the deposited material. Due to the deviations in weighing the aniline monomer for the polymerisation solution, the anodic current of 1.5 mA of the first anodic peak was achieved in a different number of cycles (from 23 to 25 cycles). Therefore, the experiment was manually stopped at –0.3 V vs. Ag with a 0.1 V uncertainty. After the deposition, the electrode was cleaned with methanol (Sigma Aldrich) to remove the residual monomer/electrolyte suspension and the unattached polymeric parts.

### 2.2. Material Characterisation

#### 2.2.1. Scanning Electron Microscopy (SEM)

Field-emission scanning electron microscopy (FE-SEM) was used to characterise the morphology of the pure-gold SPE (Au-SPE) and the electrodeposited PANI (PANI-Au-SPE). The specimens for the FE-SEM observations (Jeol JSM-7600F) were prepared by placing the samples on a conductive tape. Cross-section samples were cut perpendicular to the electrode. The Au-SPE surface was observed at an acceleration voltage of 20 kV and the PANI-Au-SPE samples at 5 kV.

#### 2.2.2. Fourier-Transform Infrared (FTIR) Spectroscopy

Fourier-transform infrared (FTIR) spectra of the prepared PANI-Au-SPE were measured using an ATR (attenuated total reflection) crystal with an ATR FTIR Frontier L1280018 spectrometer (Perkin Elmer, Waltham, MA, USA). A spectrum was obtained by sampling 20 interferograms with a resolution of 4 cm^−1^ in the range 500–4000 cm^−1^.

#### 2.2.3. Profilometer

Three-dimensional (3D) mapping of the surface roughness of the Au-SPE and PANI-Au-SPE was carried out with a Stylus profilometer DEKTAK (Bruker) having a resolution 0.3 nm in the x, 1 µm in the y, and 0.1 nm in the z directions.

### 2.3. Sensing Characterisation

**Figure 2:**
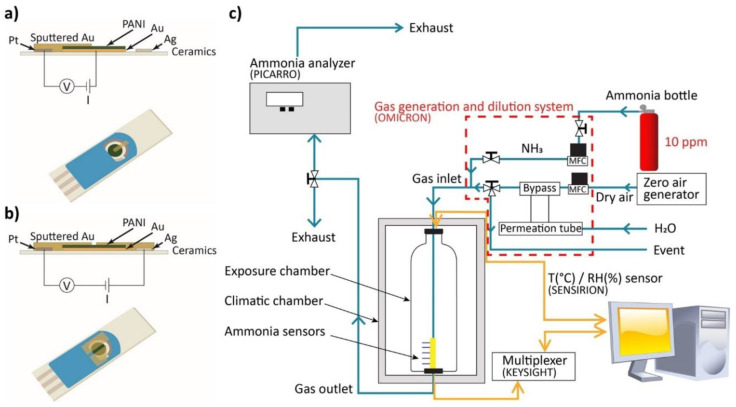
(**a**) Scheme of connection 1; (**b**) scheme of connection 2; (**c**) schematic view of measurement system for sensing.

The prepared PANI-Au-SPE samples were sputtered with gold (Agar Auto Sputter Coater from Agar Scientific) using a 3D-printed template to create an appropriate connection between the separated working, counter and reference electrodes. Two different geometries were used. Connection 1 is composed of one 2-mm-wide electrode deposited on the surface to connect the polymer surface to the counter electrode (Figure 2a). The electric field is oriented longitudinally. For connection 2, two parallel electrodes are deposited on the surface of the polymer with a 1-mm gap in between (Figure 2b), connecting the polymer to the platinum and silver parts of the SPE. A transversal electric field was applied between the counter and the reference electrodes. By using a custom-made connector for the SPE, the PANI-Au-SPE sensors were put in a dynamic exposure chamber, in which the temperature and relative humidity (RH) were controlled for all the experiments: T = 25 °C (RT) and RH for ammonia (NH_3_) sensing fixed at 70% and for humidity characterisation varying between 10 and 90%. When the sensor was exposed to the NH_3_ and humidity, a variation in the resistance of the active PANI material was measured. The behaviour of the sensor in the presence of NH_3_ is presented as a percentage of the relative response Equation (1):

(1) $$Response\;\left(\%\right)=\;\left[\frac{\left(R_{t}-R_{0}\right)}{R_{0}}\right]\cdot 100$$

where *R_t_* is the resistance at a certain time, and *R*_0_ is the initial resistance under zero air. The resistance of the sensor was measured continuously with a computerised digital multimeter (Agilent 34970A) as a function of the NH_3_/humidity concentration and the exposure time. The NH_3_ concentration was measured at the gas outlet of the gas chamber with the NH_3_ analyser (Picarro G2103) and the humidity was measured directly in the chamber with the temperature/RH sensor (Sensirion SHT25). The desired analyte concentration in the chamber was controlled by the flow rate of the two gases (NH_3_ gas and humid air) using a gas generation and dilution system (Omicron technologies OMI-SR042A-A). The complete measuring setup is presented in Figure 2c.

For the characterisation of the effect of humidity, the sensor was exposed to a certain percentage of relative humidity for 30 min in both directions (from 0% to 70% RH and back). The sensor’s stability in purified air at 70% RH was monitored over 40 h.

The protocol to analyse the behaviour of the sensor in contact with NH_3_ involved three phases: 1. Estimation of the initial resistance of the sensor after exposing it to purified air (zero air + humid air); 2. Measuring of the sensor’s resistance during exposure to the analyte. In this phase, the sensor’s resistance changes due to the interaction of the analyte with the active PANI layer; 3. Blowing the sensor flushed with purified air (zero air + humid air), causing desorption of the analyte and recovery of the sensor’s resistance. The NH_3_ was sensed by exposing the sensor for 10 min to a particular concentration of NH_3_ gas and 50 min to purified gas for the desorption. Then, for simulating real-time measurements without an intermediate process of desorption, the NH_3_ gas concentration changed every 10 min. The response time is defined as the time it takes for the sensor to reach 90% of the response change.

## 3. Results and Discussion

### 3.1. Electrodeposition of Polyaniline (PANI)

**Figure 3:**
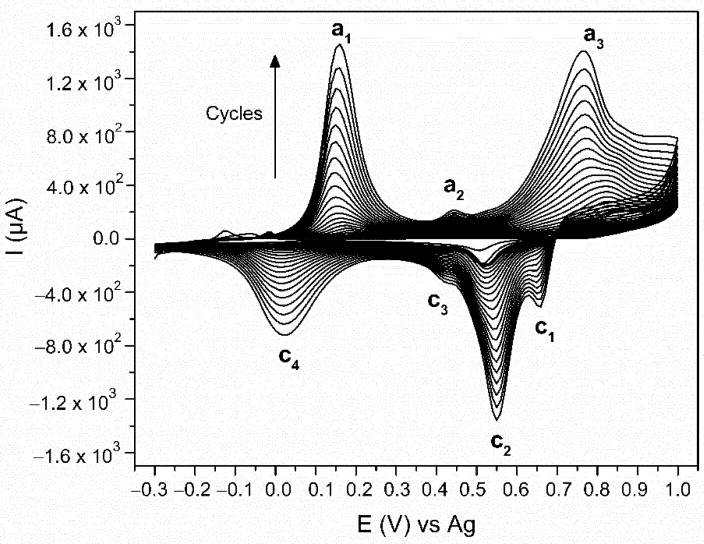
Voltammogram of aniline electropolymerisation via cyclic voltammetry (CV) on the surface of gold screen-printed electrode (Au-SPE) in HCl electrolyte.

The PANI was electrodeposited with CV using a 0.1 M aniline monomer suspension in a 1 M HCl electrolyte. Figure 3 shows all 23 cycles of the electropolymerisation process. There are three peaks observed in the anodic scan (E (a_1_) = 0.16 V, E (a_2_) = 0.45 V, and E (a_3_) 0.77 V vs. Ag) and four in the cathodic scan (E (c_1_) = 0.66 V, E (c_2_) = 0.55 V, E (c_3_) = 0.42 V, E (c_4_) = 0.02 V vs. Ag). At the beginning of the electropolymerisation, the CV is performed on the pure Au-SPE, causing Au oxidation (E = 1 V) and reduction (E = 0.51 V) [57,58]. The monomer aniline oxidation, which is the first step in the aniline electropolymerisation occurs at E = 0.9 V [43] in the anodic scan. These later peaks, however, are not visible due to the gradually increasing currents of the peaks, characteristic for the PANI’s formation and its redox behaviour. The anodic peak a_1_ is the oxidation peak of the leucoemeraldine (the fully reduced state of PANI), which oxidises to emeraldine (half-oxidised state). The anodic peak a_2_ is related to the formation of the intermediate degradation products (p-benzoquinone) and their oxidation. The anodic peak a_3_ is the oxidation of emeraldine to pernigraniline (fully oxidised state) [55,59,60]. All these processes are reversible during the cathodic scan. The cathodic peaks c_1_ and c_2_ are the reduction of pernigraniline. The splitting of the peak is associated with the acidity of the electrolyte [59]. The cathodic peak c_3_ is the reduction of the intermediate degradation products, p-benzoquinone to hydroquinone, and the cathodic peak c_4_ is the reduction of emeraldine to leucoemeraldine [55,59,60]. Leucoemeraldine is known to be an unstable state of PANI [61]; therefore, after exposing the electrode to air at the end of the electropolymerisation, it is oxidised in the direction of emeraldine. The same conclusion was made after a correlative study of the electrodeposition and electrochromic behaviour of PANI [54]. Due to the fact that we are performing the electropolymerisation in an HCl electrolyte [43], the final product is HCl-doped PANI.

### 3.2. Characterisation and Analysis of the Electrodeposited PANI

**Figure 4:**
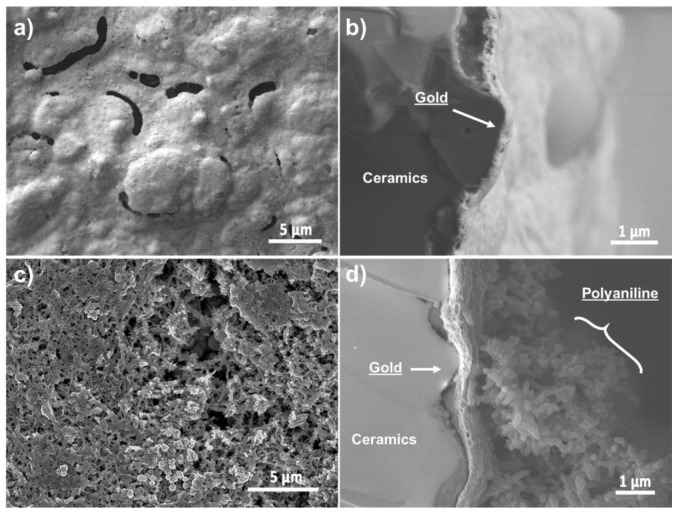
Au-SPE field-emission scanning electron microscopy (FE-SEM-LEI) images: (**a**) top view and (**b**) cross-section view; PANI-Au-SPE FE-SEM-SEI images: (**c**) top view and (**d**) cross-section.

The morphology of the Au-SPE and the PANI-Au-SPE in the form of FE-SEM images is presented in Figure 4. The Au-SPE top-view surface (Figure 4a) appears to be a layer with an uneven surface relief and larger, randomly distributed pores. From the cross-section view (Figure 4b) the Au layer takes on the surface relief of the ceramic grains of the SPE substrate. The Au layer’s thickness is approximately 250 nm. Figure 4c,d shows the top-view surface and the cross-section of the PANI-Au-SPE. The PANI electrodeposited with CV appears to be a compact film composed of interconnected nanofibers attached to the Au surface. The PANI layer’s thickness was estimated to be around 5 µm.

**Figure 5:**
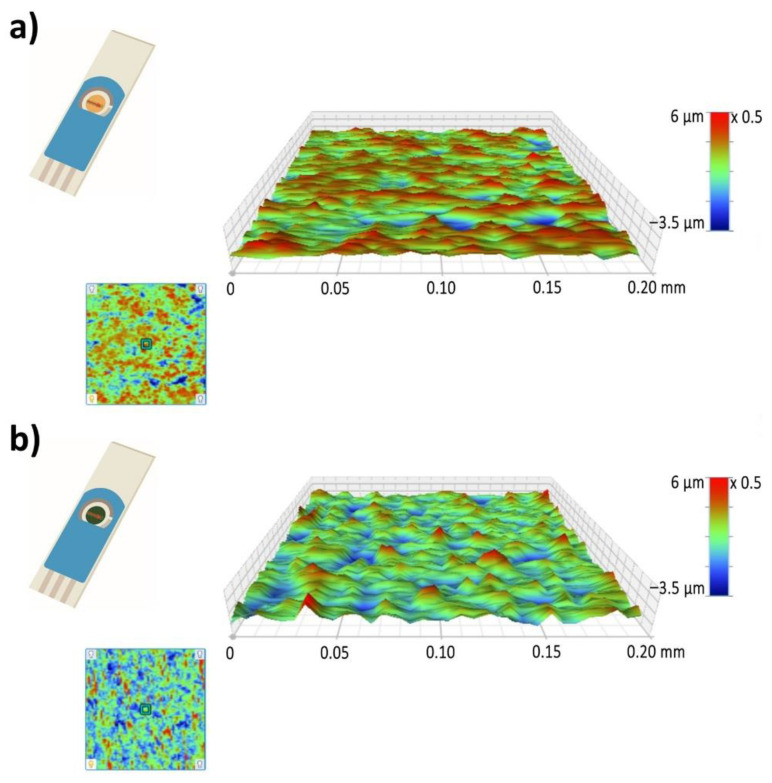
Three-dimensional (3D) mapping of surface area: (**a**) Au-SPE and (**b**) PANI-Au-SPE.

Figure 5a,b presents the 3D surface mapping of the Au-SPE and PANI-Au SPE. A 0.2 × 0.2 mm^2^ surface area was examined in the direction perpendicular to the electrode. The Au-SPE surface has an uneven relief with some randomly distributed, low areas, which is consistent with the Au-SPE SEM surface images (Figure 4a). The roughness factor (Ra) for the Au-SPE surface is approximately 0.58 µm. In the case of the PANI-Au-SPE, the Ra increases to 0.91 µm. The surface relief has fewer higher areas, but more lower areas with an open PANI structure, as seen from the PANI-Au-SPE SEM surface image (Figure 4c). Materials with a higher roughness have abundant surface-adsorption sites, causing an increased response to gas analytes [62]. Therefore, by electrodepositing the PANI with CV, we have successfully completed the first step towards obtaining suitable material for gas sensing, due to the open nanostructure of the PANI and the increased roughness.

**Figure 6:**
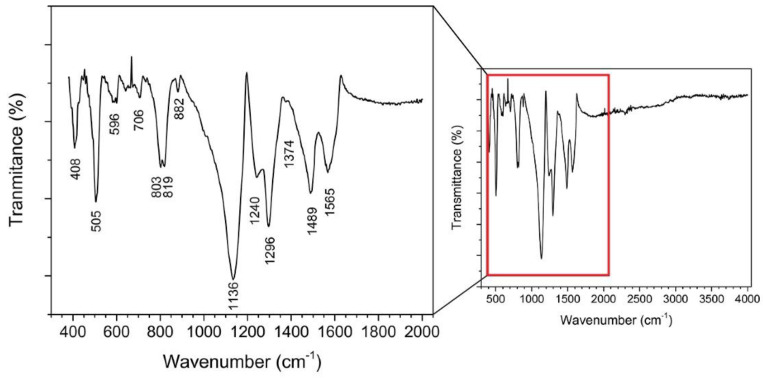
Fourier-transform infrared (FTIR) spectrum of PANI-Au-SPE with magnified fingerprint (500–1500 cm^−1^) and double bond (1500–2000 cm^−1^) region.

Fourier-transform infrared (FTIR) spectra were used to characterise the state of the PANI-Au-SPE (Figure 6). The band at 1565 cm^−1^ corresponds to the quinoid-ring stretching vibrations, while the band at 1489 cm^−1^ corresponds to the benzenoid-ring stretching vibrations. The bands at 1374 and 1296 cm^−1^ correspond to the C–N stretching in the neighbourhood of the quinoid ring and to the C–N stretching of a secondary aromatic amine. The band at 1240 cm^−1^ corresponds to the C–N^+•^ stretching vibrations in the conductive protonated form of PANI. The broad band at approximately 1136 cm^−1^ corresponds to the C–H aromatic in-plane deformation vibrations [9,63,64,65]. The bands at approximately 800 cm^−1^ originate from the C–H out-of-plane bending in the 1,4-disubstituted ring structures. These indicate the head-to-tail coupling of the aniline during the polymerisation [30,66]. This type of coupling is known for polymerisation in acidic conditions [59]. The band at 505 cm^−1^ corresponds to the bending vibrations of the PANI backbone due to the presence of a chloride ion [66], which confirms the spontaneous doping of the PANI during electropolymerisation. From the ratio of the area of the peaks corresponding to the quinoid (1565 cm^−1^) and benzenoid rings (1489 cm^−1^) (Q/B), the oxidation level can be estimated [65,67]. The oxidation level of the PANI-Au-SPE is 0.8, which is consistent with the electrochemically synthesised PANI via chronoamperometry (polymerisation at constant potential) [65]. The structural form of the emeraldine state has one quinoid ring vs. three benzenoid rings, giving a ratio of 0.75. The FTIR results confirm the formation of a conductive PANI layer on the SPE in the form of an emeraldine salt, which was expected based on the observed green colour of the polymerised electrode.

### 3.3. Impact of Humidity on the Resistance of the PANI-Au-SPE

The PANI-Au-SPE samples were firstly prepared for a two-point measuring system. The electrochemical SPE-based system has three separate electrodes, a large gap between them (approximately 1 mm) and the electrodeposition of PANI only on the surface of the gold working electrode. Therefore, a suitable connection between the working and counter electrodes (connection 1, Figure 2a) and the counter, working and reference electrode (connection 2, Figure 2b), was created with Au sputtering. The as-prepared PANI-Au-SPE sensors were characterised for the effect of humidity and NH_3_ sensing.

**Figure 7:**
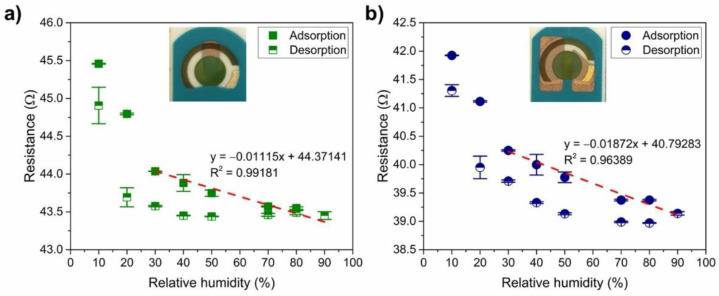
Reversibility of the PANI-Au-SPE sensor after exposing to increasing relative humidity (RH): (**a**) connection 1 and (**b**) connection 2. Both figures, *(***a**,**b**), contain linear fitting for the adsorption of water between 30% and 90% RH.

Figure 7 shows the typical response of the PANI to humidity for both types of connection (olive circle—connection 1, and navy square—connection 2): the water molecules cause a higher degree of doping, i.e., a lower resistivity by facilitating the proton exchange inside the PANI. The enhancement of the charge transport comes from the transfer from the reduced units (NH_2_^+^) to the oxidised units (NH^+^=) [40,68] and/or the increase of the ionic conduction due to the release of a proton from the water molecules that are adsorbed [69]. Both PANI-Au-SPE sensors have a similar initial resistance (the same exponential range) and show a linear trend (R^2^ > 0.96 for both connections) in the decreasing resistance with increased RH from 30% to 90% (adsorption of humidity). The difference in the sensitivity to RH (slope of the linear curve; –0.01 Ω%^−1^ for connection 1, and –0.02 Ω%^−1^ for connection 2) is two-times higher in the case of the sensor with connection 2.

Next, the reversibility of the PANI-Au-SPE sensors’ resistance after exposing them to 90% RH was investigated. Figure 7 shows the adsorption and desorption of water vapour in humid air for the PANI-Au-SPE sensor with connection 1 (Figure 7a) and connection 2 (Figure 7b). After continuous exposure to a higher RH, the PANI-Au-SPE did not return to its initial resistance at a certain RH. The desorption resistances are different from the adsorption resistances by 0.14–2.46% for connection 1 and 0.98–2.83% for connection 2. In addition to the “protonation effect”, water molecules at high RH can cause swelling of the PANI fibres, which increases the packing disorder [37] and can contribute to the irreversibility. As the PANI acts as water-sensitive material, all further measurements were performed at a constant 70% RH to eliminate the influence of the environment.

**Figure 8:**
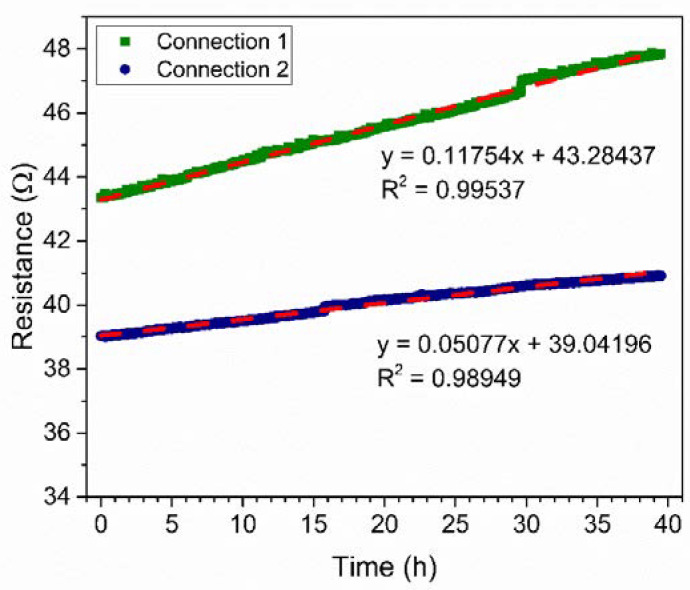
PANI-Au-SPE sensor stability at 70% of RH and room temperature (RT) for connection 1 (olive) and connection 2 (navy).

The stability of the PANI-Au-SPE was investigated by exposing the PANI-Au-SPE sensors to environmental conditions (room temperature-RT, 70% RH), which were later used for the NH_3_ detection (Figure 8). The PANI-Au-SPE sensor with both connection geometries has a linear increase in the resistance with time (R^2^ = 0.99 for both connections). Connection 1 has a two-times-higher aging effect than connection 2 (slope of the linear curve; 0.1 Ωh^−1^ for connection 1, and 0.05 Ωh^−1^ for connection 2). Thus, the PANI-Au-SPE sensor with connection 1 is more stable with time. As seen in Figure 7, connection 2 has a two-times-higher sensitivity towards RH and a two-times-lower stability with time (Figure 8). Both connections have approximately the same PANI surface area sputtered with Au, i.e., 4 mm^2^. The main difference is in the distribution of the current flows across the device, which are difficult to determine precisely given its structure.

### 3.4. NH_3_-Sensing Performance of the PANI-Au-SPE

**Figure 9:**
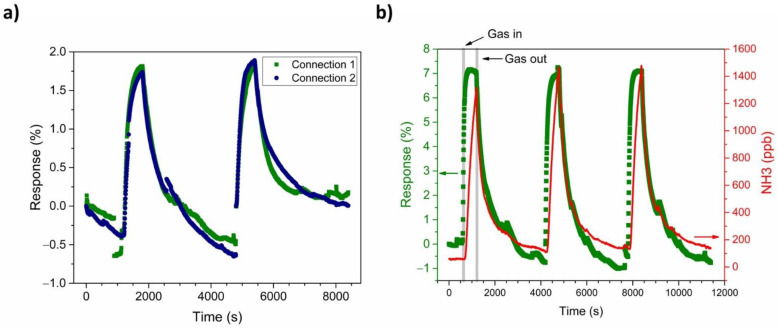
(**a**) Comparison between connection 1 (olive) and connection 2 (navy) of PANI-Au-SPE sensor response to 500 ppb NH3 and (**b**) the dynamics of PANI-Au-SPE-Au resistance change (olive) and NH3 concentration variation monitored by the PICARO analyser (red curve).

For a comparison of the influence of the connection geometry on the sensor’s response to NH_3_, PANI-Au-SPE sensors with both connections were exposed to 500-ppb NH_3_ at RT and 70% RH (Figure 9a). Both connections exhibit an equivalent relative response to the NH_3_ under the same conditions. Even though connections 1 and 2 have differences in terms of humidity characterisation (Figure 7) and sensor stability (Figure 8), they both show linear behaviour. As the PANI-Au-SPE sensor’s behaviour towards the NH_3_ is presented as a relative response, it is not influenced by this difference. Therefore, we can conclude that the current flow through the PANI film due to the geometry of the connections does not influence the PANI’s response to NH_3_ gas, which suggests the uniformity of the electrodeposited PANI.

As both connections show the same response, either could be used for the subsequent experiments. Our next results, in terms of sensor dynamics, calibration and the simulation of real-time measurements, are presented for connection 1.

**Figure 10:**
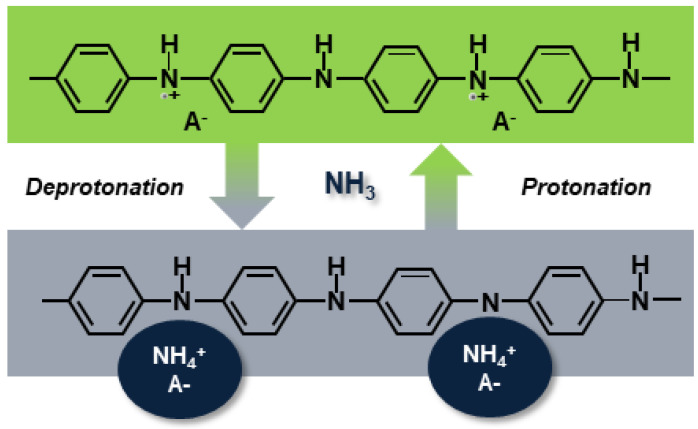
Mechanism of deprotonation/protonation at the origin of the PANI’s sensitivity to ammonia gas. A^−^, NH_3_ and NH_4_^+^ refer to the dopant, ammonia and ammonium cation, respectively.

Figure 9b shows the repeatability of the PANI-Au-SPE sensor upon exposure to 1400 ppb of NH_3_ operating at RT and 70% RH. The olive squares represent the relative PANI-Au-SPE sensor’s response and the red curve, the variation in the NH_3_ concentration at the same time, monitored by the analyser. The PANI-Au-SPE shows the character of a typical p-type semiconductor [70,71], as its resistance increases upon exposure to NH_3_. The NH_3_ sensing by the PANI follows the well-known deprotonation/protonation mechanism [25,26]. Polyaniline becomes conductive with the addition of dopants to the polymeric chain, which generates protonation on the nitrogen atom and consequently facilitates the movements of valence electrons, causing hopping conduction. In the presence of NH_3_ gas, a proton is removed from the polymeric chain to form an energetically favourable ammonium cation (NH_4_^+^), compensated in the structure by the dopant (A^−^). Removing a proton from the PANI reduces the amount and the mobility of the charge carrier, resulting in an increased resistance of the PANI [6]. In the absence of NH_3_, NH_4_^+^ decomposes to NH_3_ and H^+^, causing the reversible protonation of the PANI (Equation (2) and Figure 10) [25,26].

PANIH^+^ + NH_3_ = PANI + NH_4_^+^ (2)

Compared with the NH_3_ analyser data (Figure 9b), the PANI-Au-SPE sensor follows the behaviour of real-time NH_3_ concentration, without reaching the saturation point at the maximum NH_3_ concentration, i.e., 1400 ppb. The PANI-Au-SPE sensor shows repeatability during three cycles of exposure to NH_3_, having enough time to complete the NH_3_ desorption (50 min), and then to respond equally to the NH_3_ exposure (10 min).

**Figure 11:**
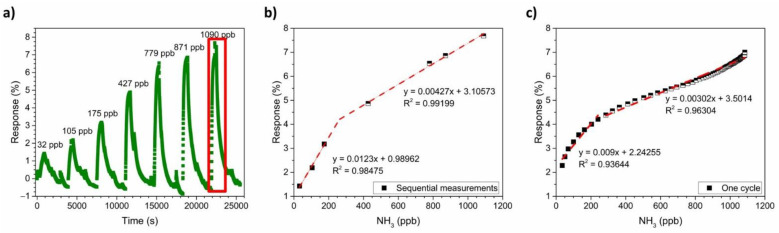
NH_3_ calibration: (**a**) PANI-Au-SPE sensor’s response after exposing to different concentrations of gas; (**b**) calibration curve obtained from the PANI-Au-SPE sensor’s response at maximum achieved gas concentration in sequential measurements and (**c**) calibration curve obtained from one cycle of measurements.

To obtain the calibration curve of the PANI-Au-SPE sensor for NH_3_, a series of NH_3_ gases with concentrations ranging from 32 ppb to 1090 ppb were used. Figure 11a shows the PANI-Au-SPE sensor’s response to different real-time NH_3_ concentrations, monitored by the analyser. A higher concentration of NH_3_ has a higher PANI-Au-SPE sensor response. By plotting the maximum sensor response vs. the NH_3_ concentration, the calibration plot for the PANI-Au-SPE sensor is obtained (Figure 11b). The PANI-Au-SPE sensor shows two linear ranges, from 32 ppb to 250 ppb (R^2^ = 0.98) and from 250 ppb to 1100 ppb (R^2^ = 0.99). The sensitivity of the PANI-Au-SPE sensor to NH_3_ detection at 70% RH and RT is 12.3% ppm^−1^ for the first linear range, and 4.27% ppm^−1^ for the second. Due to the synchronic NH_3_ concentration monitoring by the analyser, the calibration plot for the PANI-Au-SPE sensor was also made by plotting the responses of the PANI-Au-SPE sensor for the last cycle of 1090 ppb NH_3_ (Figure 11a) vs. NH_3_ analyser data at the same time (Figure 11c). The analyser gives more extensive and detailed NH_3_ data for all the measurement times. Therefore, the calibration plot of one cycle has more points in comparison to the calibration plot made from the maximum responses of sequential measurements (Figure 11b). The calibration plot of one cycle (Figure 11c) has the same two linear ranges, from 32 ppb to 250 ppb (R^2^ = 0.94) and from 250 ppb to 1100 ppb (R^2^ = 0.96) with a similar sensitivity (9% ppm^−1^ for the first linear range, and 3.02% ppm^−1^ for the second). As both ways of obtaining the calibration plot provide the same result, the calibration obtained by applying data from the analyser simplifies the sensor’s characterisation, i.e., a larger number of points made with only one measurement. The theoretical detection limit is calculated according to the formula from IUPAC recommendations Equation (3):

(3) $$LOD\;\left(ppb\right)=\;\frac{3\sigma }{m},$$

where *σ* is the standard deviation of the sensing 15-min baseline before the NH_3_ exposure and m is the slope of the first calibration curve (0.0123% pbb^−1^). The detection limit is calculated to be 23 ppb, which is a realistic number regarding to the lowest detected concentration (quantification limit 32 pbb).

**Figure 12:**
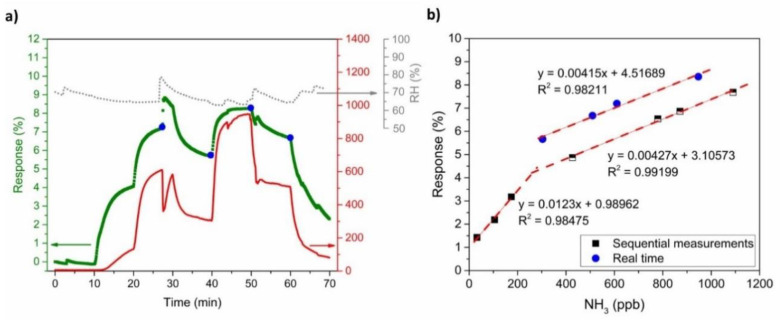
(**a**) PANI-Au-SPE sensor’s response, NH_3_ concentration, and RH vs. time—simulation of real-time measurement and (**b**) comparison of PANI-Au-SPE sensor’s responses obtained from real-time measurement with the calibration curve.

In a real-time environment the sensors are not exposed to absorption and desorption cycles, but the gas concentration varies continuously without a recovery time. Figure 12a shows the PANI-Au-SPE sensor response (olive squares) to a continuously changing NH_3_ concentration (red curve). The PANI-Au-SPE sensor follows the same trend as the NH_3_ concentration, proving that the system can have the same response trend without any additional recovery time. The out-of-range jump in the sensor’s response and the NH_3_ analyser at 27 min is a consequence of the RH instability (grey curve) at the same time. It was already proven [30] that different humidity conditions influence the PANI’s sensitivity to NH_3_, due to the reaction between water and NH_3_. Thus, it is important to maintain constant humidity conditions in the case of NH_3_ sensing or to monitor simultaneously the humidity to correct the signal. For a comparison of the real-time sensor responses with measurements from a typical laboratory cycle, four points (blue dots in Figure 12a) were inserted into the calibration plot, acquired from sequential measurements (Figure 11b), and are presented in Figure 12b. All the real-time points represent a linear trend in the higher NH_3_ concentration range (250 ppb to 1100 ppb) with the same sensitivity.

Table 1 summarises the characteristics of the PANI-Au-SPE sensor constructed and studied here with state-of-the-art PANI NH_3_ sensors at RT [8,30,70,71,72,73]. All the presented PANI systems from the literature were based on a chemically synthesised PANI and without the presence of any nanomaterials. Indeed, the PANI/HCl from a chemical synthesis shows a quantification limit at 1000 ppb [73] when the electropolymerised PANI/HCl from this investigation has a quantification limit 31 times lower. Regarding the response time, we also need to consider preliminary experiments with the reference NH_3_ analyser, showing a slow response to the real-time NH_3_ concentration increase inside the gas chamber. Because the concentration is not that high, it is impossible to distinguish the response time from the actual filling time. Thus, the calculated response time for the presented system is over-estimated and is probably much shorter.

Regarding the PANI’s′ conductivity and sensing performance, the addition of an appropriate nanomaterial, i.e., electron donors or acceptors, in the form of metal, metal oxide nanoparticles or carbon nanotubes, enhances the conductivity due to the p-n heterojunction at the interface between the p-type PANI and n-type nanoparticles [33,34,50]. If the PANI’s sensing characteristic is changed to n-type, p-n heterojunctions are formed by the addition of material, which acts as p-type (electron acceptor), e.g., carbon nanotubes [35]. As a result, ultra-low detection limits (0.25 ppb at 60% RH and RT [50], 0.25 ppb [33] and <16 ppb [34] at RT with humidity compensation) can be achieved. In contrast, this work is based on pure PANI with the simplest dopant (HCl) and no additional nanomaterial. Nevertheless, due to the film’s morphology and low thickness, a sufficiently low quantification limit (32 ppb) and good performance (sensitivity: 12.3% ppm^−1^ [32–200 ppb] and 4.27% ppm^−1^ [200–1000 ppb]) were achieved. Comparing the presented sensing system based on PANI with the literature on PANI for NH_3_ sensing as presented in Table 1, better sensing properties in terms of sensitivity were found, mainly due to the addition of complex dopants and nanomaterials. Our proposed sensing system is comparable to the best of the state-of-the-art literature for PANI/NH_3_ sensors; however, it offers the advantages of ease of fabrication and simplicity where PANI is doped only with an univalent ion (Cl^−^). At this stage, any further work on adding nanomaterials to the system has the potential to improve sensitivity. With the good baseline of the current sensor, the addition of nanomaterials could, in the first stage, be focused on adding a certain material that could catalyse the target analyte to produce NH_3_ and enable the indirect detection of other toxic compounds.

In the field of sensors, four main characteristics make a material/system suitable: sensitivity, repeatability, reproducibility, and selectivity. As shown in the calibration plots (Figure 11a,b) and by comparing with the literature (Table 1), the PANI-Au-SPE has a high sensitivity in the low-ppb range. Moreover, the reproducibility and the repeatability of our PANI-Au-SPE was proven by comparing electrodes with different connection geometries (Figure 9a) and by performing several continuous cycles (Figure 9b). Finally, as the selectivity of PANI for NH_3_ detection was already proven [21,26,30,71], our PANI-Au-SPE sensor is an excellent example of an NH_3_ sensor system. Generally, after comparing our work with previously reported studies [6,8,30,70,71,72,73] we can conclude that electrochemically deposited PANI is an under-examined but up-and-coming area of NH_3_ gas sensing. Due to its practical and controllable preparation via electropolymerisation and SPE’s direct use, the proposed procedure reveals novel aspects for NH_3_ sensing.

## 4. Conclusions

We have shown that electrochemically synthesised PANI doped with HCl can be successfully deposited on a gold, screen-printed electrode (Au-SPE) and used as an efficient room-temperature NH_3_-gas sensor. With our approach, we have successfully bridged the gap from 3-electrode electrochemical systems suitable for PANI electrodeposition to 2-electrode resistivity systems suitable for NH_3_ gas sensing, which was further achieved via additional Au sputtering. Such prepared PANI-Au-SPE sensors show exceptionally good repeatability, reproducibility and sensitivity in the low-ppb NH_3_ range. The incorporation of the gas analyser as an additional component for checking and controlling the target gas inlet contributes to the system characterisation, i.e., fewer measurements for a calibration curve with multiple points, enable real-time sensor responses and genuine results, i.e., monitoring the sensor’s responses during gas interflow and not only at the maximum concentration. Electrochemical synthesis is considered to be unconventional as it is rarely used in gas sensing, but this fully controllable way of preparing PANI, and its combination with a three-electrode SPE system, leads to novel aspects in PANI gas-sensing systems.

## Figures and Tables

**Table 1:** Metrological parameters for the NH_3_ gas sensors based on PANI at RT.

Material	PANI Synthesis	Detection Limit ^1^ [ppb]	Quantification Limit ^2^ [ppb]	Sensitivity [% ppm^−1^]	Response Time [min]
PU-PANI (CSA) [8]	Chemical	/	20	0.8	5 at 1 ppm
PBuA-PANI (HCl) [8]	Chemical	/	250	10	2.5 at 1 ppm
PVDF-PANI (DBSA) [8]	Chemical	/	100	17	2.5 at 1 ppm
WO3@PANI [71]	Chemical	3	500	/	4.2 for >0.25 ppm
PANI (H_2_SO_4_) [30]	Chemical	7	/	15	3 at 50 ppm
PANI-MWCNT (HCl) [70]	Chemical	/	200	/	1.3 at 12 ppm
PANI (DBSA) ^3^ [72]	Chemical	/	40	7900	/
PANI (HCl) [73]	Chemical	/	1000	/	/
PANI (HCl) [this work]	Electrochemical	23	32	12.3 [32–200 ppb] & 4.27 [200–1000 ppb]	5.2 at 1 ppm

^1^ Theoretical detection limit. ^2^ Lowest measured concentration. ^3^ Results were only reported as an evolution of relative impedance and not the relative resistance.

## Data Availability

Data sharing not applicable.
